# Early predictors of poor outcome after out-of-hospital cardiac arrest

**DOI:** 10.1186/s13054-017-1677-2

**Published:** 2017-04-13

**Authors:** Louise Martinell, Niklas Nielsen, Johan Herlitz, Thomas Karlsson, Janneke Horn, Matt P. Wise, Johan Undén, Christian Rylander

**Affiliations:** 1 Department of Anaesthesiology and Intensive Care Medicine, Institute of Clinical Sciences, Sahlgrenska Academy, University of Gothenburg, SE-413 45 Gothenburg, Sweden; 2 Department of Clinical Sciences, Lund University, Lund, Sweden; 3 The Centre for Pre-hospital Research in Western Sweden, University College of Borås and Sahlgrenska University Hospital, Gothenburg, Sweden; 4 Health Metrics at Sahlgrenska Academy, University of Gothenburg, Gothenburg, Sweden; 5 Department of Intensive Care, Academic Medical Centre, University of Amsterdam, Amsterdam, The Netherlands; 6 Adult Critical Care, University Hospital of Wales, Cardiff, UK; 7 Department of Intensive Care and Perioperative Medicine, Lund University, Malmö, Sweden

**Keywords:** Out-of-hospital cardiac arrest, Intensive care, Prognosis, Risk score

## Abstract

**Background:**

Early identification of predictors for a poor long-term outcome in patients who survive the initial phase of out-of-hospital cardiac arrest (OHCA) may facilitate future clinical research, the process of care and information provided to relatives. The aim of this study was to determine the association between variables available from the patient’s history and status at intensive care admission with outcome in unconscious survivors of OHCA.

**Methods:**

Using the cohort of the Target Temperature Management trial, we performed a post hoc analysis of 933 unconscious patients with OHCA of presumed cardiac cause who had a complete 6-month follow-up. Outcomes were survival and neurological function as defined by the Cerebral Performance Category (CPC) scale at 6 months after OHCA. After multiple imputations to compensate for missing data, backward stepwise multivariable logistic regression was applied to identify factors independently predictive of a poor outcome (CPC 3–5). On the basis of these factors, a risk score for poor outcome was constructed.

**Results:**

We identified ten independent predictors of a poor outcome: older age, cardiac arrest occurring at home, initial rhythm other than ventricular fibrillation/tachycardia, longer duration of no flow, longer duration of low flow, administration of adrenaline, bilateral absence of corneal and pupillary reflexes, Glasgow Coma Scale motor response 1, lower pH and a partial pressure of carbon dioxide in arterial blood value lower than 4.5 kPa at hospital admission. A risk score based on the impact of each of these variables in the model yielded a median (range) AUC of 0.842 (0.840–0.845) and good calibration. Internal validation of the score using bootstrapping yielded a median (range) AUC corrected for optimism of 0.818 (0.816–0.821).

**Conclusions:**

Among variables available at admission to intensive care, we identified ten independent predictors of a poor outcome at 6 months for initial survivors of OHCA. They reflected pre-hospital circumstances (six variables) and patient status on hospital admission (four variables). By using a simple and easy-to-use risk scoring system based on these variables, patients at high risk for a poor outcome after OHCA may be identified early.

## Background

For patients who are unconscious after out-of-hospital cardiac arrest (OHCA) and treated with hypothermia, survival rates around 50% are reported [1, 2]. Pre-hospital factors such as time from collapse to start of cardiopulmonary resuscitation (CPR), time from collapse to return of spontaneous circulation (ROSC), initial rhythm, bystander CPR and lactate levels all are strongly correlated with outcome at a group level [2–7]. However, these correlations are based on large retrospective cohorts with no control for differences in patient treatment and may therefore be subject to bias. Furthermore, single predictors may not be reliable in individual cases, owing to difficulties in obtaining or recording precise information during pre-hospital management of cardiac arrest (CA) [8, 9]. Currently, neurological prognostication in patients remaining unconscious is not recommended earlier than 72 h after the CA [10]. Nevertheless, in the OHCA patient population, with its elevated mortality, an earlier prediction of poor outcome is desirable for the process of care and information provided to the patient’s relatives. An assessment of the risk for a poor outcome would also be of great value for comparing populations and to define patient risk when assessing effects in interventional studies. Efforts to construct a prediction score based on the set of data available at hospital admission have yielded the OHCA score [11] and the Cardiac Arrest Hospital Prognosis (CAHP) score [12]. When applied prospectively, the OHCA score showed moderate predictive accuracy [13]; however, none of these scores has been validated for clinical use.

The aim of this study was to establish a clinically useful association between parameters available from patient history and status at intensive care admission and outcome in comatose survivors of OHCA. The first step in the analysis was directed at determining the association between these factors and poor outcome at 6 months, defined as Cerebral Performance Category (CPC) 3–5 [14]. The second step was directed at constructing an easy-to-use risk score for prediction of a poor outcome.

## Methods

We performed a post hoc analysis of data obtained in the Target Temperature Management (TTM) trial [15], in which researchers recruited patients from 36 intensive care units (ICUs) in Europe and Australia. The trial included adult patients (≥18 years) resuscitated from OHCA of a presumed cardiac cause who remained unconscious (Glasgow Coma Scale [GCS] score ≤8) more than 20 minutes after ROSC. The main exclusion criteria were unwitnessed asystole as the initial rhythm and refractory shock at hospital admission defined as sustained systolic blood pressure less than 80 mmHg despite administration of fluids, vasopressors, inotropes and/or treatment with an intra-aortic balloon pump or left ventricular assist device [16].

Pre-hospital data, including initial rhythm, witnessed arrest, administration of bystander CPR and time from collapse to ROSC, were systematically collected at admission according to the Utstein guidelines [17]. Time from CA to initiation of basic life support (BLS; administered by bystanders or first responders) and advanced life support (ALS) was recorded. No-flow and low-flow times were defined as the time from CA to the start of CPR (BLS or ALS) and the time from the start of CPR to ROSC, respectively. Time to ROSC was defined as the time from CA to the first recorded time point of sustained spontaneous circulation. Patients were included in the present analysis if their CPC was recorded at follow-up 6 months after CA. All sites participating in the TTM trial registered patient data in a common electronic case report form. The process was monitored at each site by external reviewers who visited the centres and verified the correctness of registered data. All the centres used the same study protocol that defined target temperature management over time and prompted multimodal investigations for neurological prognostication. The results of the main trial were subjected to sensitivity analyses for time, study centre and other possible biases, all of which turned out negative.

The TTM trial demonstrated no difference in mortality and neurological outcome between a target temperature of 33 °C and 36 °C. The result has been further elaborated in post hoc analyses and sub-studies, which have so far shown similar outcomes in the two target temperature groups [18–21]. Therefore, data were pooled for the present analysis.

### Statistical analysis

A description of original data is given in Tables 1, 2 and 3, where categorical variables are presented as crude numbers and percentages and continuous variables are presented as medians with 25th and 75th percentiles. Logistic regression was used to calculate age-adjusted ORs with corresponding 95% CIs and *p* values. Continuous variables not fulfilling the linearity assumption were transformed using either natural logarithm or square root transformation.

**Table 1:** Patient characteristics in relation to outcome

	Outcome at 6 months		Adjusted for age^a^	
	CPC 1–2 (*n* = 440)	CPC 3–5 (*n* = 493)	OR (95% CI)^b^	*p* Value
Age, years	61 (52–69)	68 (61–76)	1.06 (1.05–1.07)	<0.0001
Female sex	66 (15)	111 (23)	1.69 (1.18–2.43)	0.004
BMI (5/20)^c^	25.3 (23.4–27.8)	26.1 (23.9–29.4)	1.05 (1.01–1.08)	0.006
Alcoholism (0/2)	10 (2)	26 (5)	3.12 (1.45–6.71)	0.004
COPD/asthma (0/1)	31 (7)	65 (13)	1.62 (1.01–2.58)	0.04
Previous heart failure (0/3)	16 (4)	44 (9)	1.75 (0.95–3.22)	0.07
Diabetes (2/4)	51 (12)	89 (18)	1.41 (0.96–2.08)	0.08
CABG (0/4)	26 (6)	62 (13)	1.49 (0.90–2.44)	0.12
TIA/stroke (1/3)	23 (5)	50 (10)	1.51 (0.89–2.57)	0.13
Malignancy (3/4)	9 (2)	22 (4)	1.89 (0.82–4.35)	0.13
Epilepsy (1/1)	11 (3)	5 (1)	0.46 (0.15–1.47)	0.19
Arrhythmia (0/2)	60 (14)	103 (21)	1.26 (0.87–1.83)	0.21
ICD (3/1)	1 (<1)	4 (<1)	3.77 (0.40–35.44)	0.25
Hypertension (1/3)	150 (34)	222 (45)	1.15 (0.86–1.52)	0.35
Dialysis (0/1)	2 (<1)	4 (<1)	1.78 (0.32–10.10)	0.51
IHD (1/2)	101 (23)	157 (32)	1.10 (0.80–1.50)	0.57
Intravenous drug abuse (2/1)	2 (<1)	2 (<1)	1.67 (0.21–13.04)	0.63
Pacemaker (2/2)	11 (3)	21 (4)	1.17 (0.54–2.52)	0.68
Cardiac valve surgery (1/4)	10 (2)	15 (3)	1.17 (0.50–2.73)	0.71
PCI (0/4)	45 (10)	62 (13)	1.08 (0.70–1.65)	0.73
Previous cardiac arrest (0/2)	9 (2)	12 (2)	1.13 (0.44–2.91)	0.79
Immunodeficiency (2/1)	2 (<1)	2 (<1)	1.16 (0.14–9.25)	0.89
Previous AMI (0/2)	79 (18)	112 (23)	0.99 (0.70–1.39)	0.94
Cirrhosis (0/1)	0 (0)	3 (<1)		
AIDS (4/4)	1 (<1)	0 (0)		

*Abbreviations: CPC* Cerebral Performance Category, *BMI* Body mass index, *COPD* Chronic obstructive pulmonary disease, *CABG* Coronary artery bypass grafting, *TIA* Transient ischaemic attack, *ICD* Implantable cardioverter defibrillator, *IHD* Intermittent haemodialysis, *PCI* Percutaneous coronary intervention, *AMI* Acute myocardial infarction, *AIDS* Acquired immune deficiency syndrome, immunodeficiency other than AIDS

Data are presented as number (%) or median (25th and 75th percentiles)

Poor outcome was defined as CPC 3–5 at 6 months

^a^Except for age itself

^b^OR for poor outcome (CPC 3–5) with corresponding 95% CI

^c^Number missing in the two groups, respectively

**Table 2:** Circumstantial factors

	Outcome at 6 months		Adjusted for age	
	CPC 1–2 (*n* = 440)	CPC 3–5 (*n* = 493)	OR (95% CI)^a^	*p* Value
CA at home (0/1)^b^	192 (44)	306 (62)	2.16 (1.64–2.85)	<0.0001
Witnessed (0/1)	406 (92)	427 (87)	0.61 (0.39–0.97)	0.04
Bystander CPR (0/2)	347 (79)	331 (67)	0.61 (0.45–0.83)	0.002
First monitored rhythm other than VT/VF (0/1)	38 (9)	169 (34)	5.14 (3.46–7.64)	<0.0001
Intubation (6/8)	273 (63)	352 (73)	1.67 (1.24–2.25)	0.0007
CA until BLS (73/141)	1 (0–2)	1 (0–3)	1.10 (1.04–1.17)	0.0005
CA until ALS (8/8)	8 (5–11)	10 (7–15)	1.07 (1.04–1.10)	<0.0001
CA until ROSC (0/1)	20 (14–30)	31 (21–47)	1.57 (1.42–1.72)^c^	<0.0001
No flow^d^ (2/4)	1 (0–3)	2 (0–8)	1.09 (1.05–1.12)	<0.0001
Low flow^e^ (0/1)	19 (12–27)	27 (17–40)	1.43 (1.31–1.56)^c^	<0.0001
Adrenaline (3/2)	258 (59)	423 (86)	4.62 (3.29–6.48)	<0.0001
Seizures (1/2)	33 (8)	23 (5)	0.64 (0.36–1.15)	0.14
Automatic compression (0/3)	92 (21)	127 (26)	1.27 (0.92–1.75)	0.15

*Abbreviations: CA* Cardiac arrest, *CPC* Cerebral Performance Category, *CPR* Cardiopulmonary resuscitation, *VT/VF* Ventricular tachycardia/ventricular fibrillation, *BLS* Basic life support, *ALS* Advanced life support, *ROSC* Return of spontaneous circulation

Data are presented as number (%) or median (25th and 75th percentiles)

Poor outcome defined as CPC 3–5 at 6 months

^a^OR for poor outcome (CPC 3–5) with corresponding 95% CI

^b^Number missing in the two groups, respectively

^c^Square root transformed

^d^No flow: time from CA to start of CPR measured in minutes

^e^Low flow: time from start of CPR to ROSC measured in minutes

**Table 3:** Patient factors based on examination on arrival at hospital

	Outcome at 6 months			Adjusted for age	
	CPC 1–2 (*n* = 440)	CPC 3–5 (*n* = 493)	*p* Value	OR (95% CI)^a^	*p* Value
Initial temperature (12/23)^b^	35.5 (34.9–36.0)	35.3 (34.4–36.0)	0.002	0.11 (0.03–0.47)	0.003
Pupillary/corneal reflex (8/36)	392 (91)	327 (72)	<0.0001	0.27 (0.18–0.40)	<0.0001
Cough reflex (45/59)	277 (70)	211 (49)	<0.0001	0.41 (0.30–0.55)	<0.0001
Spontaneous breathing (15/22)	310 (73)	284 (60)	<0.0001	0.57 (0.43–0.77)	0.0002
GCS-M1 (1/6)	173 (39)	316 (65)	<0.0001	2.79 (2.11–3.70)	<0.0001
pH (24/20)	7.27 (7.17–7.32)	7.19 (7.05–7.28)	<0.0001	0.02 (0.01–0.06)	<0.0001
Lactate (36/25)	4.6 (2.4–8.1)	6.9 (3.9–10.6)	<0.0001	1.84 (1.55–2.20)^c^	<0.0001
Creatinine (13/17)	95 (80–115)	115 (90–140)	<0.0001	3.36 (2.14–5.29)^d^	<0.0001
Blood glucose (32/20)	12.4 (9.4–16.0)	14.0 (10.6–18.0)	<0.0001	1.08 (1.06–1.12)	<0.0001
PaCO_2_ (31/25)			0.0006		0.0004
<4.5 kPa	40 (10)	66 (14)		1.96 (1.22–3.16)	
>6.0 kPa	191 (47)	256 (55)		1.75 (1.29–2.38)	
Base excess (40/26)	−6 (−10, −4)	−10 (−15, −5)	<0.0001	0.93 (0.91–0.95)	<0.0001
Potassium (15/13)	3.7 (3.4–4.2)	4.0 (3.5–4.5)	<0.0001	1.55 (1.29–1.87)	<0.0001
Shock at admission (0/1)	36 (8)	100 (20)	<0.0001	2.70 (1.77–4.13)	<0.0001
PaO_2_ > 40 kPa (35/35)	64 (16)	95 (21)	0.07	1.38 (0.96–2.00)	0.09
FiO_2_, % (11/20)	80 (50–100)	90 (53–100)	0.21	1.00 (1.00–1.01)	0.25
Acute STEMI/LBBB (2/8)	217 (50)	220 (45)	0.21	0.96 (0.73–1.26)	0.77

*Abbreviations: CPC* Cerebral Performance Category, *GCS-M1* Glasgow Coma Scale motor score 1, *PaCO*_*2*_ Partial pressure of carbon dioxide in arterial blood, *PaO*_*2*_ Partial pressure of oxygen in arterial blood, *FiO*_*2*_ Fraction of inspired oxygen, *STEMI* ST segment elevation myocardial infarction, *LBBB* Left bundle branch block

Patients with abnormal values of PaCO_2_ (<4.5 or >6.0 kPa, respectively) were compared with patients with normal values

Data are presented as number (%) or median (25th and 75th percentiles)

Poor outcome defined as CPC 3–5 at 6 months

^a^OR for poor outcome (CPC 3–5), with corresponding 95% CI

^b^Number missing in the two groups, respectively

^c^Square root transformed

^d^Transformed by the natural logarithm

Owing to the amount of missing data for several of the variables, multiple imputation was used for the multivariable analysis. Missing data were assumed to be missing at random (*p* < 0.01 for Little’s test of missing completely at random), and 50 imputed datasets were generated with the Markov chain Monte Carlo method and using the expectation-maximisation algorithm. Rubin’s rules were used when pooling the results from the imputed datasets.

To identify independent predictors of a poor outcome, we started with a full model including all variables in Tables 1, 2 and 3. We excluded some continuous variables from the statistical analysis because of collinearity: CA-BLS time, CA-ALS time, CA-ROSC time, lactate, blood glucose and base excess on arrival to the hospital, as well as the dichotomous variables, intravenous drug abuse, immunodeficiency, cirrhosis and AIDS, that were present in fewer than five cases. Multiple logistic regression was performed in each of the 50 imputed datasets, and the variable with the highest *p* value in the pooled result was excluded from the model. A new regression analysis was then performed in each imputed dataset, and of the remaining variables, the one with the highest *p* value in the pooled result was excluded. This procedure was repeated until all remaining variables yielded a *p* value below 0.01 in the pooled result. These variables were then used to develop our prognostic risk score (TTM risk score). To facilitate clinical use of the model, we used an approach similar to that adopted in the development of the Framingham Risk Score [22]. We let the increase in risk associated with a 5-year increase in age, reflected by five times the β-coefficient for age in the final model, correspond to 1 point. We then determined points associated with each of the other categories of the identified risk factors by how far in regression units each category was from the corresponding factor’s base category (i.e., when points = 0), dividing that distance by five times the β-coefficient for age and rounding to the nearest integer.

The AUC was used to evaluate discrimination and the concordance percentage, and the Hosmer-Lemeshow goodness-of-fit test was used to evaluate calibration. This was done for all 50 imputed datasets, and the median and range of these are presented. The first five imputed datasets were used to calculate predicted versus observed risk as well as sensitivity, specificity, and positive and negative predicted values. Youden’s J statistic was used to select the optimum cut-off of the score. Internal validation was also performed in the first five imputed datasets using bootstrapping (1000 resamples in each set), and the maximum of these was used as an estimate of optimism. No external validation was performed.

Two-sided tests were used, and *p* values below 0.01 were considered statistically significant. All analyses were performed using SAS version 9.3 for Windows software (SAS Institute, Cary, NC, USA), except for Little’s test, for which IBM SPSS Statistics version 23 software (IBM, Armonk, NY, USA) was used.

## Results

A total of 939 patients were included in the original TTM trial. Neurological outcome data 6 months after CA were available for 933 (99%) patients, who consequently were included in the present analysis. Of these patients, 493 (52%) had a poor outcome of CPC 3–5. Their median age was 65 years (IQR 57–73 years), and 177 (19%) were female.

### Variables associated with a poor outcome

Older age, female sex, higher body mass index and a previous history of alcoholism were associated with a poor outcome (Table 1). Longer time from collapse to arrival of providers of BLS and ALS, as well as initial rhythm other than ventricular tachycardia or ventricular fibrillation (VT/VF), was associated with a poor outcome. CA location at home, longer duration of no flow and low flow, increasing time to ROSC and pre-hospital intubation and administration of adrenaline were also associated with a poor outcome (Table 2).

Lower pH, base excess and initial body temperature were associated with a poor outcome. Absence of brainstem reflexes, such as spontaneous breathing as well as pupillary, corneal and cough reflexes, were also associated with a poor outcome. Associated with a poor outcome were higher lactate; initial shock; higher glucose, potassium and creatinine in plasma; and absence of motor response at pain stimulation (Glasgow Coma Scale motor score 1 [GCS-M1]). Abnormal partial pressure of carbon dioxide in arterial blood (PaCO_2_; <4.5 or >6.0 kPa) in initial blood gas samples was also associated with a poor outcome (Table 3).

### Independent predictors of a poor outcome

Six variables were excluded from the multivariate analysis because of multicollinearity: CA to BLS and CA to ALS were highly correlated to no flow, and CA to ROSC to low flow and lactate, blood glucose and base excess were all correlated to pH. After stepwise backward elimination, ten variables remained significantly correlated to a poor outcome: older age, CA occurring at home, initial rhythm other than VF/VT, longer duration of no flow, longer duration of low flow, treatment with adrenaline, bilateral absence of corneal and pupillary reflexes, GCS-M1, a lower pH and a PaCO_2_ lower than 4.5 kPa on admission (Table 4). This final model showed good discrimination with a median (range) AUC of 0.844 (0.842–0.846) and a median AUC of 0.820 by internal validation with bootstrap-derived samples correcting for optimism. The Hosmer-Lemeshow goodness-of-fit test demonstrated overall good calibration with *p* > 0.40 for all 50 imputations.

**Table 4:** Independent predictors of Cerebral Performance Category 3–5 at 6 months

	OR (95% CI)	*p* Value	β- Coefficient
Age, years	1.07 (1.05–1.08)	<0.0001	0.0644
CA at home	1.75 (1.26–2.44)	0.0008	0.5620
First monitored rhythm other than VT/VF	4.06 (2.55–6.46)	<0.0001	1.4014
No flow, minutes	1.06 (1.02–1.10)	0.002	0.0578
Low flow, minutes	1.28 (1.15–1.42)^a^	<0.0001	0.2430
Treatment with adrenaline	2.08 (1.39–3.11)	0.0003	0.7342
No pupillary or corneal reflex	2.46 (1.58–3.85)	<0.0001	0.9016
pH	0.10 (0.03–0.34)	0.0003	2.3308
GCS motor score 1^b^	2.00 (1.44–2.77)	<0.0001	0.6927
PaCO_2_ < 4.5 kPa	2.53 (1.46–4.39)	0.0009	0.9293

*Abbreviations: CA* Cardiac arrest, *GCS* Glasgow Coma Scale, *PaCO*_*2*_ Partial pressure of carbon dioxide in arterial blood, *VT/VF* Ventricular tachycardia/ventricular fibrillation

^a^Square root-transformed values

^b^GCS motor score 1 = no motor reaction to a painful stimuli

### A simple risk score for a poor outcome: performance and validation

The TTM risk score was developed using the final selection of variables above. The points assigned to different variables are listed in Table 5. The minimum sum of points was −2, and the maximum was 35. The performance of the TTM score using quartiles as cut-offs is described in Table 6. The median (range) AUC was 0.842 (0.840–0.845), and corrected for optimism by internal validation it was 0.818 (0.816–0.821). The Hosmer-Lemeshow goodness-of-fit test yielded a *p* value >0.10 in all 50 imputations, showing good calibration. The median concordance percentage was 82.2 (range 81.0–82.5). In patients with a score above 13 points, the sensitivity for poor outcome was 69% to 70% with a corresponding specificity of 83% to 84%.

**Table 5:** Target temperature management risk score points (range −2 to 35)

Risk factor	Categories	Points
Age, years	<40	−1
	40–44	0
	45–49	1
	50–54	2
	55–59	3
	60–64	4
	65–69	5
	70–74	6
	75–79	7
	80–84	8
	≥85	9
CA at home	No	0
	Yes	2
First monitored rhythm other than VT/VF	No	0
	Yes	4
No flow	0–4	0
	5–9	1
	10–14	2
	≥15	3
Low flow	0–5	0
	6–15	1
	16–30	2
	31–60	3
	>60	4
Treatment with adrenaline	No	0
	Yes	2
No pupillary or corneal reflex	No	0
	Yes	3
pH	≥7.35	−1
	7.20–7.34	0
	7.05–7.19	1
	6.90–7.04	2
	<6.90	3
GCS motor score 1	No	0
	Yes	2
PaCO_2_ < 4.5 kPa	No	0
	Yes	3

*Abbreviations: CA* Cardiac arrest, *GCS* Glasgow Coma Scale, *PaCO*_*2*_ Partial pressure of carbon dioxide in arterial blood, *VT/VF* Ventricular tachycardia/ventricular fibrillation

Points assigned to categories of the ten independent risk factors for a poor outcome in the calculation of the Target temperature management risk score. Poor outcome was defined as Cerebral Performance Category 3–5 at 6 months after out-of-hospital cardiac arrest. Total score ranged from −2 to 35. No flow is defined as time from CA to start of cardiopulmonary resuscitation in minutes. Low flow is defined as time from start of cardiopulmonary resuscitation to return of spontaneous circulation in minutes

**Table 6:** Discrimination performance of the three risk scores

Risk score	Scores		
TTM risk score	>10	>13	>16
*n* (%)	612–615 (66–66)	410–417 (44–45)	217–221 (23–24)
Sensitivity, %	86–87	69–70	40–41
Specificity, %	57–58	83–84	95–96
PPV, %	69–70	82–83	91–91
NPV, %	79–80	71–71	59–59
CAHP risk score	>150	>200	
*n* (%)	711–716 (76–77)	312–318 (33–34)	
Sensitivity, %	91–91	48–49	
Specificity, %	39–40	82–83	
PPV, %	63–63	76–76	
NPV, %	79–79	59–59	
OHCA risk score	>2.0	>17.4	>32.5
*n* (%)	829–833 (89–89)	565–574 (62–62)	266–271 (29–29)
Sensitivity, %	94–94	77–78	44–45
Specificity, %	16–17	56–58	89–89
PPV, %	56–56	66–67	82–82
NPV, %	71–73	69–70	59–59

*Abbreviations: CAHP* Cardiac Arrest Hospital Prognosis, *NPV* Negative predictive value, *PPV* Positive predictive value, *ROSC* Return of spontaneous circulation, *TTM* Target Temperature Management trial

Discrimination performance of the three different risk scores in our TTM trial cohort with minimum-maximum values of the first five imputations. The TTM risk score is divided into quartiles where the Youden’s J statistic cut-off (>13 points) coincides with second quartile upper limit (i.e., the median). The CAHP and OHCA risk scores are calculated and divided as described in their respective original publications [11, 12]. They were defined as high risk if >200 points and >32.5 points for the CAHP and OHCA risk scores, respectively

### Risk for a poor outcome assessed by OHCA and CAPH scores

On the basis of the formula presented by Adrie et al. [11], we calculated the OHCA risk score in our cohort for each of the 50 imputations. This rendered a median (range) AUC of 0.746 (0.739–0.752), and 48 of the 50 imputations (96% of the imputed datasets) had a *p* value <0.05 for the Hosmer-Lemeshow goodness-of-fit test, indicating poor calibration. Median (range) concordance was 74.4 (73.8–75.1). For patients with a high risk according to the original paper (score >32.5), the sensitivity was 44% to 45% and the specificity was 89% to 89% for a poor outcome.

By visually estimating the parameters in the risk score model presented by Maupain et al. [12], we also calculated the CAHP risk score in our cohort for each of the 50 imputations, yielding a median (range) AUC of 0.746 (0.743–0.747), and 20 (40%) of the 50 imputations had *p* values <0.05 for the Hosmer-Lemeshow goodness-of-fit test, indicating poor calibration. The median (range) concordance percentage was 74.3 (74.0–74.6). In patients with a score above 200 points, which Maupain et al. defined as high risk, the sensitivity for poor outcome was 48% to 49% with a corresponding specificity of 82% to 83%.

**Fig. 1:**
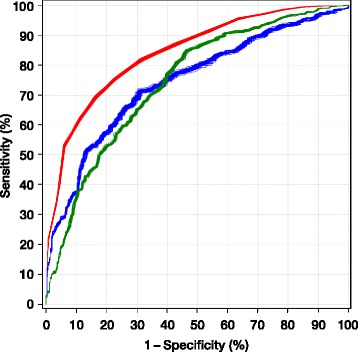
Comparison of the performance of the Target Temperature Management (TTM), out-of-hospital cardiac arrest (OHCA) and Cardiac Arrest Hospital Prognosis (CAHP) risk scores. ROC curves for a poor outcome at 6 months for the TTM (*red*), OHCA (*blue*) and CAHP (*green*) risk scores in our imputed sample (one curve per score for each of the 50 imputations). The median AUC for the TTM score was 0.842 (0.818 corrected for optimism); for the OHCA score, it was 0.746, and for the CAHP score, it was 0.746

The performance of the OHCA and CAHP scores is shown in Table 6. A comparison of the performance using ROC curves of the three risk scores is presented in Fig. 1.

## Discussion

The aim of this study was to identify independent parameters from patient history and status available at intensive care admission that could be used for early prediction and risk stratification of a poor outcome in comatose survivors following OHCA. Age, time to ROSC and initial temperature were previously reported from the TTM cohort [21, 23, 24]. Our findings using multiple variable analysis are also in line with earlier studies that older age, CA occurring at home, initial rhythm other than VF/VT, longer duration of no flow, longer duration of low flow, treatment with adrenaline, absence of corneal and pupillary reflexes, GCS-M1, a lower pH, and a PaCO_2_ lower than 4.5 kPa on admission were independent predictors of a poor outcome [2, 6, 11, 18–20, 25–36]. Low-flow time from the initiation of CPR to ROSC has previously been associated with a poor outcome in various studies of OHCA [11, 29, 37], including the present cohort [21]. However, effective low flow may have been longer than registered because BLS was provided before arrival of ALS in 73% of the patients. Notwithstanding that BLS efficacy may vary considerably and is difficult to assess, registered low-flow time in the present study was somewhat longer than reported in the cohorts used to calculate OHCA and CAHP risk scores [11, 12]. The role of adrenaline in the resuscitation of OHCA victims is controversial, and in a recent meta-analysis, no benefit could be found [30]. In a Japanese study of 400,000 patients, researchers reported increased ROSC but decreased 1-month survival in patients who received adrenaline compared with those who did not [31]. Adrenaline is still part of European resuscitation guidelines [38], and it is possible that the association between adrenaline and a poor outcome that we found was related to more complicated resuscitations. Among variables potentially available at ICU admission, co-morbidity may be expected to be important. However, as in this analysis, it has previously been shown not to be associated with mortality in the present patient cohort [39].

To assess the risk for poor outcome after OHCA at an early stage, we constructed the TTM risk score on the basis of individual variables available at ICU admission. Several prediction methods have previously been investigated. The Acute Physiology and Chronic Health Evaluation II score [40], which is not disease-specific, has repeatedly been shown to be a poor predictor of outcome in OHCA [41]. The OHCA risk score presented in 2006 using variables available at hospital admission showed a similar performance. However, the OHCA risk score was based on a small cohort (*n* = 130). Also, the patients were relatively young compared with other OHCA cohorts, with a median age of 55 years and no patients older than 69 years [11]. The recently published CAHP risk score, based on a large number of patients, has performed best so far with an AUC of 0.93. However, some steps in its underlying calculations, such as the assumption of a linear association between outcome and time to ROSC, as well as outcome and pH, can be discussed [12]. When applied in our cohort, the OHCA and the CAPH scores lost performance as assessed by AUC, sensitivity, specificity, positive predictive value (PPV) and negative predictive value (NPV). These discrepancies reflect the problem of low external validity for scores that are constructed in a single cohort. This may also apply to our score in spite of our use of a multicentre international cohort, and it needs to be validated in further trials. For the TTM risk score, a high value (>16 points, representing the fourth quartile of the patients) showed a promising PPV of 91% and a specificity of 95% to 96% and therefore indicated an acceptable margin not to predict a poor outcome in a patient with a good prognosis. However, the corresponding sensitivity was only 43%. Selecting a performance optimum cut-off of >13 points by use of Youden’s J statistics, yielding a sensitivity and specificity of 69% to 70% and 83% to 84%, respectively, does not render the score more useful, because the PPV is reduced to 82% to 83%. A low sensitivity when reaching a satisfactory specificity was also an issue for the CAHP risk score (sensitivity 46% to 56% in the development cohort). The high-risk cut-off OHCA score at 32.5 points (development cohort) described in the paper had an inadequate specificity of only 77% for poor outcome but a better sensitivity of 77% [11]. These results provoke the question whether better performance could be achieved with more, or contrarily, with fewer but highly discriminative, variables in a risk score model. Interestingly, Jabre et al. [42], using a development cohort of 1771 patients, found 0% (95% CI 0.0% to 0.5%) survival in 772 patients who fulfilled three criteria during pre-hospital resuscitation. These criteria were (1) OHCA not witnessed by emergency medical services personnel, (2) non-shockable initial cardiac rhythm and (3) no ROSC before receipt of a third 1-mg dose of epinephrine. However, comparison with our TTM trial cohort is difficult because the latter included only patients hospitalised alive after OHCA. Among 618 patients admitted alive in the Jabre et al. study, 22% were discharged alive. There is no information about the 180-day survival, which was close to 50% in the TTM cohort [15].

### Strengths and limitations

A significant advantage of the present study is the well-defined cohort of patients who were carefully evaluated. In particular, on one hand, a major strength was that patients in the TTM trial were subject to strict rules on neurological prognostication and withdrawal of life-sustaining therapy [15], which may be a significant source of bias in cohort studies and other randomised clinical trials. On the other hand, our risk score is valid only under these clinical conditions. A large number of clinically relevant variables and outcome measures were registered in similar pre-hospital and emergency health care systems and ICUs following a published trial protocol [16]. This may favour evaluation of heterogeneous early predictors of outcome [9]. Treating all patients included in a randomised trial of two interventions as one cohort carries a principal risk of hiding differences between the groups. However, the two groups were well balanced with regard to background characteristics and the main outcome, and outcomes in numerous sub-studies have shown no differences due to the intervention. Nevertheless, it needs to be stressed that a major limitation of the external validity of our study is that the cohort consists of selected patients with OHCA of a presumed cardiac origin who were not found in unwitnessed asystole, who were unconscious on admission to hospital and who were not in a refractory circulatory shock state. Furthermore, the modality or the degree of respiratory support before ICU admission constitutes a treatment bias because it was not registered in this study and could have affected predictors such as PaCO_2_ and pH.

## Conclusions

In a cohort of 933 patients with OHCA of a presumed cardiac cause extracted from the 939 patients included in the TTM trial, we found ten independent predictors of a poor outcome, defined as CPC 3–5 six months after CA. These included older age, CA occurring at home, initial rhythm other than VT/VF, longer duration of no flow, longer duration of low flow, administration of adrenaline, bilaterally absent pupillary and corneal reflexes, absent motor response to pain, a lower pH, and a PaCO_2_ lower than 4.5 kPa at admission.

The predictors readily available at ICU admission were used to construct an easy and simple-to-use risk score that showed good association with outcome 6 months after the arrest. The score could further represent a helpful tool for treatment allocation and stratification in randomised studies as well as for comparison of cohorts in epidemiological studies. However, it is important to stress that the proposed risk score is not yet precise enough to be used for individual prognostication of outcome after OHCA, and it needs further validation in a large cohort of patients with OHCA.

## Abbreviations

ALS: Advanced life support
AMI: Acute myocardial infarction
BLS: Basic life support
BMI: Body mass index
CA: Cardiac arrest
CABG: Coronary artery bypass grafting
CAHP: Cardiac Arrest Hospital Prognosis
COPD: Chronic obstructive pulmonary disease
CPC: Cerebral Performance Category
CPR: Cardiopulmonary resuscitation
FiO_2_: Fraction of inspired oxygen
GCS: Glasgow Coma Scale
GCS-M1: Glasgow Coma Scale motor score 1
ICD: Implantable cardioverter defibrillator
ICU: Intensive care unit
IHD: Intermittent haemodialysis
LBBB: Left bundle branch block
NPV: Negative predictive value
OHCA: Out-of-hospital cardiac arrest
PaCO_2_: Partial pressure of carbon dioxide in arterial blood
PaO_2_: Partial pressure of oxygen in arterial blood
PCI: Percutaneous coronary intervention
PPV: Positive predictive value
ROSC: Return of spontaneous circulation
STEMI: ST segment elevation myocardial infarction
TIA: Transient ischaemic attack
TTM: Target Temperature Management trial
VT/VF: Ventricular tachycardia/ventricular fibrillation
